# Partial wavelet coherence as a robust method for assessment of neurovascular coupling in neonates with hypoxic ischemic encephalopathy

**DOI:** 10.1038/s41598-022-27275-8

**Published:** 2023-01-10

**Authors:** Tim Hermans, Katherine Carkeek, Anneleen Dereymaeker, Katrien Jansen, Gunnar Naulaers, Sabine Van Huffel, Maarten De Vos

**Affiliations:** 1 Department of Electrical Engineering (ESAT), STADIUS, KU Leuven, Leuven, Belgium; 2 Department of Development and Regeneration, KU Leuven, Leuven, Belgium; 3 Neonatal Intensive Care Unit, UZ Leuven, Leuven, Belgium; 4 Neonatal Intensive Care Unit, Cliniques Universitaires Saint Luc, Brussels, Belgium; 5 Child Neurology, UZ Leuven, Leuven, Belgium

**Keywords:** Biomedical engineering, Neonatal brain damage, Data processing, Predictive markers

## Abstract

In neonates with hypoxic ischemic encephalopathy, the computation of wavelet coherence between electroencephalogram (EEG) power and regional cerebral oxygen saturation (rSO2) is a promising method for the assessment of neurovascular coupling (NVC), which in turn is a promising marker for brain injury. However, instabilities in arterial oxygen saturation (SpO2) limit the robustness of previously proposed methods. Therefore, we propose the use of partial wavelet coherence, which can eliminate the influence of SpO2. Furthermore, we study the added value of the novel NVC biomarkers for identification of brain injury compared to traditional EEG and NIRS biomarkers. 18 neonates with HIE were monitored for 72 h and classified into three groups based on short-term MRI outcome. Partial wavelet coherence was used to quantify the coupling between C3–C4 EEG bandpower (2–16 Hz) and rSO2, eliminating confounding effects of SpO2. NVC was defined as the amount of significant coherence in a frequency range of 0.25–1 mHz. Partial wavelet coherence successfully removed confounding influences of SpO2 when studying the coupling between EEG and rSO2. Decreased NVC was related to worse MRI outcome. Furthermore, the combination of NVC and EEG spectral edge frequency (SEF) improved the identification of neonates with mild vs moderate and severe MRI outcome compared to using EEG SEF alone. Partial wavelet coherence is an effective method for removing confounding effects of SpO2, improving the robustness of automated assessment of NVC in long-term EEG-NIRS recordings. The obtained NVC biomarkers are more sensitive to MRI outcome than traditional rSO2 biomarkers and provide complementary information to EEG biomarkers.

## Introduction

Perinatal asphyxia is a complication where blood flow to the neonatal brain is restricted during labour or delivery. The corresponding lack of oxygen supply to the brain may induce brain injury, namely hypoxic-ischemic encephalopathy (HIE). Neonates with the suspicion or confirmation of perinatal asphyxia and signs of encephalopathy are admitted to the neonatal intensive care unit (NICU) to assess the severity of HIE, minimize permanent brain injury and enhance recovery. Therapeutic hypothermia (TH) is currently considered standard clinical care in neonates with moderate and severe HIE. This therapy involves cooling the neonate to 33.5 °C for 72 h, which enhances recovery from the hypoxic insult. Early monitoring of the vulnerable neonatal brain in these first days of life is important, as it identifies neonates with different degrees of brain injury, guides medical treatment, and may suggest long-term developmental prognosis^1^.

Electro-encephalography (EEG) and near-infrared spectroscopy (NIRS) are non-invasive monitoring techniques commonly used in the NICU to continuously monitor the neonatal brain after an hypoxic insult^2^. EEG provides a continuous measure of spontaneous (background) neuronal activity of the brain. The affected brain after a hypoxic insult may reveal different abnormal background EEG patterns, with low-voltage and discontinuous traces, correlating with the severity of the hypoxic insult. Recovery of the brain is characterized by normalization of the EEG to normal-voltage, continuous tracings^3^. Over the last 45 years, numerous methods have been proposed for both visual and automated analysis of background EEG patterns in order to assist with the assessment of HIE severity^4,5^. In general, these methods aim to describe the level of suppression and continuity of the EEG^6–8^. With NIRS, cerebral blood oxygenation (rSO2) is measured, derived from relative changes in concentrations of oxygenated and deoxygenated haemoglobin^9,10^. Since perinatal asphyxia and HIE cause cerebral haemodynamic changes, several research groups have recently investigated the trends of rSO2 signals after an hypoxic insult, with literature suggesting that increased rSO2 levels are related to severe short-term and long-term outcomes^11,12^.

Although there exists a considerable amount of literature on the individual relationships between EEG and HIE, or NIRS and HIE, research focussing on the combined use of EEG and NIRS in neonates with HIE is in its infancy. The first studies combining NIRS and EEG compared the individual and combined prognostic value of EEG and NIRS biomarkers^13–20^. The majority of the studies concluded that EEG is the strongest predictor of severity, but that the combination of EEG and NIRS biomarkers have increased predicted value compared to EEG alone^13,14,16,17,19^. Later studies focussed on the relationship between EEG and NIRS features and found correlations between EEG activity and NIRS-derived parameters, indicating a coupling between cerebral activity and cerebral oxygenation^21–23^.

Whereas the aforementioned studies focussed on EEG and NIRS relationships at a global level, recent developments focussed on quantifying the dynamic coupling between EEG and NIRS signals at a more local level. Quantification of such dynamic coupling between EEG and NIRS can be interpreted as a measure for neurovascular coupling (NVC), which describes the balance between cerebral activity and cerebral blood flow (CBF)^24–26^. More specifically, NVC drives the increase in CBF in response to increased cerebral activity to meet the metabolic demands^21,27,28^. In the case of brain injury, it is hypothesized that NVC may be impaired, or inversely, that impaired NVC may induce cerebral injury due to hyper- or hypoperfusion.

Several algorithms to assess NVC in neonates have been proposed, which use EEG-derived features as a measure for cerebral activity and NIRS-derived rSO2 as a measure for changes in CBF^27^. To quantify the coupling between EEG and rSO2, a wide range of mathematical coupling measures have been proposed, such as regression analysis, radial basis function kernel and (nonlinear) transfer entropy^29–32^. In the context of HIE, Govindan et al.^24^ utilized spectral coherence to capture coupling between EEG and NIRS signals and showed increased coherence in HIE neonates with favourable outcome. To improve the analysis of the non-stationary nature of EEG and rSO2 signals, Chalak et al.^25^ and Das et al.^26^ have recently proposed a wavelet coherence method to quantify NVC and showed that decreased coherence during TH is related to adverse short- and long-term outcome.

One important assumption for using rSO2 as a surrogate for CBF is that arterial oxygen saturation (SpO2) is constant^33^. However, in long-term continuous monitoring, this assumption is not likely to be correct, especially in sick neonates. Changes in SpO2 will introduce changes in rSO2 that are not directly linked to changes in CBF, challenging the interpretation of EEG-rSO2 coupling as NVC^33,34^. Moreover, changes in SpO2 may also affect EEG, as reduced levels of arterial oxygenation could reduce cerebral metabolic activity. Therefore, SpO2 may be considered a confounding factor when analysing the coupling between EEG and rSO2 and could introduce apparent coupling between rSO2 and EEG that is not related to NVC. A limitation of the aforementioned studies that investigated NVC in HIE is that changes in SpO2 were not taken into account.

Outside the context of EEG-NIRS coupling in HIE, several methods have been proposed to correct for the relationship between SpO2 and rSO2, for example by using the fractional tissue oxygen extraction index (FTOE) instead of rSO2, or by removing changes in rSO2 that are driven by changes in SpO2 using subspace projections^31,34^. These methods can be applied as a pre-processing step, independently of the coupling function and aim to remove dynamics in the rSO2 signal that can be explained by changes in SpO2. Besides removal of influences of SpO2 on rSO2 as a pre-processing step, the removal of confounding SpO2 influence can also be incorporated in the method that computes the coupling. Considering wavelet coherence between two signals, a common method for correcting for changes of a third confounding signal is partial wavelet coherence (PWC), which has been extensively applied in various research domains, but has not yet been used in the context of NVC in HIE^35–41^.

In this study, we build further on the work of Chalak et al., and propose partial wavelet coherence as a method for NVC quantification that corrects for confounding changes in SpO2. Additionally, we put the obtained NVC biomarkers in perspective by comparing their prognostic value with traditional EEG and NIRS biomarkers in a HIE patient cohort.

## Methods

### Wavelet analysis

Wavelet analysis is a mathematical tool for analysing localised variations in time series^42^. In this study, the *continuous wavelet transform* (CWT) is used, which decomposes a signal $x$ into a time–frequency representation by convolving the signal with scaled versions of a mother wavelet^42,43^:

1 $${W}_{x}\left(s, n\right)=\sqrt{\frac{\updelta t}{s}}{\sum }_{i=1}^{N}x\left(i\right){\psi }_{0}\frac{\left(i-n\right)\delta t}{s}$$

Here, ${W}_{x}(s, n)$ is the wavelet transform of $x$, $s$ is the wavelet scale, $n$ the discrete time index, $\delta t$ is the sampling period of $x$, $x(i)$ refers to the ith time sample of $x$, and ${\psi }_{0}$ is the mother wavelet function. Throughout this study, a complex Morlet wavelet function is used with ${\omega }_{0}=6$^43^. The scales are inversely related to frequencies and for the chosen Morlet wavelet, the relation between the frequency $f$ and the wavelet scale $s$ is $f\approx \frac{1}{1.033s}$^42^. The amplitude squared of the wavelet coefficients (${|{W}_{x}(s,n)|}^{2}$) can be interpreted as the power of the corresponding frequency at timepoint $n$. In further notation we will omit the explicit dependency on $s$ and $n$, i.e., ${W}_{x} :{W}_{x}(s,n)$.

*Wavelet coherence* captures covariations between two non-stationary time series. Like the CWT, wavelet coherence is a function of scale $s$ and time $n$ and is defined as the normalized smoothed cross-wavelet transform^43,44^:

2 $${R}_{xy}\left(s, n\right)=\frac{S\left({s}^{-1}{W}_{xy}\right)}{\sqrt{S\left({s}^{-1}{\left|{W}_{x}\right|}^{2}\right)\cdot S\left({s}^{-1}{\left|{W}_{y}\right|}^{2}\right)}}$$

Here, ${W}_{xy}(s,n)={W}_{x}{W}_{y}^{*}$ is the cross-wavelet transform, where * indicates the complex conjugate. $S$ represents a smoothing function in both time and scale. In this study, smoothing in time is done by convolution with a filter given by the absolute value of the complex wavelet function (a Gaussian for the Morlet wavelet) at each scale^42,43^. Smoothing in scale is done by computing a moving average in windows equal to the number of scales in one octave (5 in this study)^43^. The smoothing function essentially implements a moving (weighted) average in time–frequency space, and hence the numerator can be interpreted as the local covariance of ${W}_{x}$ and ${W}_{y}$, whereas the terms in the denominator can be interpreted as local variances of ${W}_{x}$ and ${W}_{y}$. Consequently, ${R}_{xy}(s,n)$ represents the local correlation of two signals in time–frequency space around scale $s$ and timepoint $n$.

When reporting wavelet coherence results, typically the wavelet coherence magnitude squared $\left({\left|{R}_{xy}\right|}^{2}\right)$ and the phase $\phi$ are reported. ${\left|{R}_{xy}\right|}^{2}$ is a normalized quantity between 0 and 1 and is high at scales (frequencies) and times where signals $x$ and $y$ strongly co-vary. The angle of the cross-wavelet transform ${\phi }_{xy}(s,n)=\mathrm{arg}\left({W}_{xy}\right)$ is used to identify the phase difference between the common dynamics in $x$ and $y$^45^. This phase represents the direction of the coupling, much like the sign of a correlation coefficient indicates the direction of the correlation. If the coherence between two signals is high and $\phi \approx 0^\circ$, then the two signals move in the same direction (in-phase), whereas a phase of $\phi \approx 180^\circ$ indicates that the two signals co-vary in opposite directions (anti-phase).

When there is a third signal $z$ that has confounding effects on $x$ and $y$, a change in $z$ can result in high wavelet coherence between $x$ and $y$. If signal $z$ is known, this confounding effect can be removed by computing the *partial wavelet coherence*^36,38^:

3 $$R{P}_{xy,z}(s,n)=\frac{{R}_{xy}-{R}_{xz}{R}_{yz}^{*}}{\sqrt{\left(1-{\left|{R}_{xz}\right|}^{2}\right)}\sqrt{\left(1-{\left|{R}_{yz}\right|}^{2}\right)}}$$

Note that this formula resembles the one for partial correlation, and hence partial wavelet coherence can be interpreted as partial correlation in time–frequency space. The magnitude squared value $R{P}_{xy,z}^{2}={\left|R{P}_{xy,z}\right|}^{2}$ represents the correlation in time–frequency space between $x$ and $y$ after any linear relationship with $z$ in time–frequency space has been removed from $x$ and $y$. The phase of the partial correlation is the angle of the partial wavelet coherence: ${\phi }_{xy,z}\left(s,n\right)=\mathrm{arg}(R{P}_{xy,z}).$

### Dataset

Data was recorded at the neonatal intensive care unit of the University Hospitals of Leuven, Belgium as part of standard clinical practice according to relevant guidelines. The protocol for this retrospective anonymous data analysis was approved by the Ethics Committee Research of University Hospitals Leuven. The informed consent was waived by the ethical committee of the University Hospitals of Leuven due to the retrospective nature of the study. All data was anonymized and managed in accordance with applicable laws and regulations including the Belgian laws on privacy and patients’ rights, and the General Data Protection Regulation of the European Union. The dataset includes 18 (near)-term neonates that underwent therapeutic hypothermia (TH) who had continuous concomitant EEG, rSO2 and SpO2 monitoring in the first three days of life. Additionally, all patients had a cerebral magnetic resonance imaging (MRI) scan taken between 3 and 9 days of life. In this patient group, there were no cases of intraventricular hemorrhage or subdural hemorrhage. Table 1 gives an overview of the patient characteristics. The median time between birth and the start cooling was 105 min (IQR: 89–196). The MRIs were scored by four independent reviewers using a ‘Novel Magnetic Resonance Imaging Score’ developed by Weeke et al.^46^. The obtained MRI scores were then used to grade and categorize the outcomes of the infants with HIE as mild, moderate or severe outcome. A total MRI score of 0 was classified as a mild outcome, a score of 1 or 2 as a moderate outcome and a score greater than 2 as a severe outcome. Recordings started at a median of 133 min (IQR 12–469) after onset of TH and ended 101 h (IQR 90–110) after onset of TH. Only data recorded between onset of TH and 72 h after onset of TH were included in the analysis.

**Table 1:** Patient details.

	Mild (n = 7)	Moderate (n = 8)	Severe (n = 3)	Mild vs moderate and severe
Female	3/7	5/8	2/3	ns
GA	40.0 (39.86–40.57)	38.57 (37.43–39.15)	39.0 (38.93–39.50)	p = 0.047
Birthweight (g)	3230 (2885–3400)	3253 (3038–3783)	3570 (3510–4035)	ns
Apgar 1 min	2.0 (1.5–2.5)	0.5 (0.0–1.0)	1.0 (0.5–2.5)	p = 0.007
Apgar 5 min	4.0 (3.5–4.0)	3.5 (2.0–4.0)	1.0 (0.5–3.0)	ns
pH	6.95 (6.89–7.20)	7.13 (7.07–7.23)	6.83 (6.77–6.90)	ns

Median values are reported per outcome group and the inter-quartile range is indicated between brackets. The p-value is the Mann–Whitney U test comparing to the mild group to the pooled moderate and severe group. ns = not significant. GA: gestational age in weeks, Apgar: standardised clinical assessment of the neonate directly after birth. Low scores indicate abnormal conditions.

### Data collection and pre-processing

Multichannel EEG was recorded using nine EEG channels according to the modified international 10–20 standard. The C3–C4 bipolar derivation alone was used for analysis as this derivation is least prone to artefacts. Artefacts in the EEG were automatically identified by setting thresholds on the local standard deviation and maximum amplitude in 1 s windows, following Kota et al.^8^. The EEG was filtered with a 50 Hz notch filter and a 4th order Butterworth band-pass between 0.3 and 20 Hz. For computation of partial wavelet coherence, the EEG was further processed by computing the average band-power in 2–16 Hz using the CWT^42,47^. Subsequently, the power was averaged in non-overlapping windows of 3 s and then log-transformed to end up with an EEG power signal sampled at 1/3 Hz.

Single-channel NIRS was recorded with a neonatal INVOS sensor placed on the left or right-side of the forehead and SpO2 was recorded by means of a Masimo saturation probe on the right hand. All data were transferred to a Philips V70 monitor, which synchronized the EEG, rSO2 and SpO2 signals. The sampling rate of the rSO2 and SpO2 signals was 1 Hz. Artefacts in the rSO2 and SpO2 signals were automatically detected by an in-house algorithm checking for anomalies. This algorithm first computes a moving average of the absolute first time-derivative in moving windows of 10 s, also known as the line length, and then finds outliers of this moving line length. Artefact gaps shorter than 10 s were filled using linear interpolation. Since the signals contained virtually no information above 1/6 Hz, the signals were down-sampled to 1/3 Hz by averaging the signals in 3-s windows without overlap.

Where appropriate, samples identified as artefacts were either set to zero (e.g. when filtering) or simply ignored (e.g. when computing averages). Prior to computation of the CWT (e.g. when computing EEG power and partial wavelet coherence), additional pre-processing was done. Prior to computing the wavelet transform of a signal, the signal was de-trended by removing second-order polynomial trends and normalized to zero mean and unit standard deviation^43,44^. To compute the wavelet transform, artefacts in the signal were set to zero. After wavelet transformation, the locations of the original artefacts in the signal were used to determine locations in the time–frequency space of the resulting wavelet transform that were significantly affected by these artefacts. This was achieved by computing the total relative weight of artefact samples in the convolution of Eq. (1). Locations in the time–frequency plane for which the contribution of artefact samples was too high were marked as artefacts.

### Neurovascular coupling

To estimate NVC, we propose to compute partial wavelet coherence between log EEG power and rSO2, while removing confounding effects of SpO2. In other words, we compute Eq. (3) where $x$ is log EEG power, $y$ is rSO2 and $z$ is SpO2. A Monte Carlo analysis with 100 repetitions was employed to determine significance levels for the PWC, using the iterative amplitude adjusted Fourier transform (IAAFT) to create surrogates for EEG power, rSO2 and SpO2^43,48^. Locations in the time–frequency coherence map with PWC values exceeding 95% of the corresponding surrogate PWC values were considered significant (p < 0.05).

The partial wavelet coherence maps were further processed to condense the results. Frequency profiles were obtained by computing the percentage of time with significant PWC. Furthermore, the distribution of the phase of significant coherence values was studied to investigate the direction of the coupling. Ultimately, the percentage of time with significant PWC (NVC PWC_sig_), as well as the percentage of time with significant in-phase ($\left|\phi \right|<90^\circ )$ PWC (NVC PWC_sig,inphase_) was computed in the frequency band 0.25–1 mHz to yield biomarkers for NVC, as proposed by Chalak et al.^25^.

### EEG and NIRS biomarkers

As a reference, classical quantitative EEG and NIRS biomarkers that are sensitive to short-term outcome were computed. For EEG analysis, the pre-processed EEG was divided into non-overlapping 4-s segments to compute the power spectral density (PSD) using the periodogram method. From the PSD the total EEG power (EEG TP) in the 0.5–19.5 Hz frequency band was computed, as well as the spectral edge frequency (EEG SEF), defined as the frequency below which 95% of the total power is located^6^. Ultimately the median TP and SEF were computed across segments. For NIRS analysis, the median rSO2 value was computed (rSO2 median), as well as the percentage of values below 55% or above 90% (rSO2 abnormal)^12,20^.

### Analyses

We analysed NVC, EEG and NIRS in four different time windows: 0–24 h, 24–48 h, 48–72 h and 0–72 h, where 0 h refers to the time when cooling was started. These time windows therefore represent each day of cooling and the total cooling period. The analysis consists of three parts. First, partial wavelet coherence results obtained during the entire cooling period (0–72 h) were investigated in terms of frequency profiles and phase. Secondly, the NVC, EEG and NIRS biomarkers were compared between the three outcome groups for each of the four time windows. Here, the Mann–Whitney U test was applied to test for differences between the mild group vs the pooled moderate and severe group. Ultimately, we investigated whether the NVC biomarkers contain complementary information to EEG and NIRS markers (computed in 0–72 h) by computing their correlation and visualising the relationship between EEG SEF, NVC PWC_sig_ and MRI outcome.

## Results

### Dataset

Out of the 18 neonates included, seven neonates were classified as having a mild adverse outcome based on early MRI, eight as a moderate outcome and three as a severe outcome. Table 1 presents an overview of the patient details.

**Figure 1:**
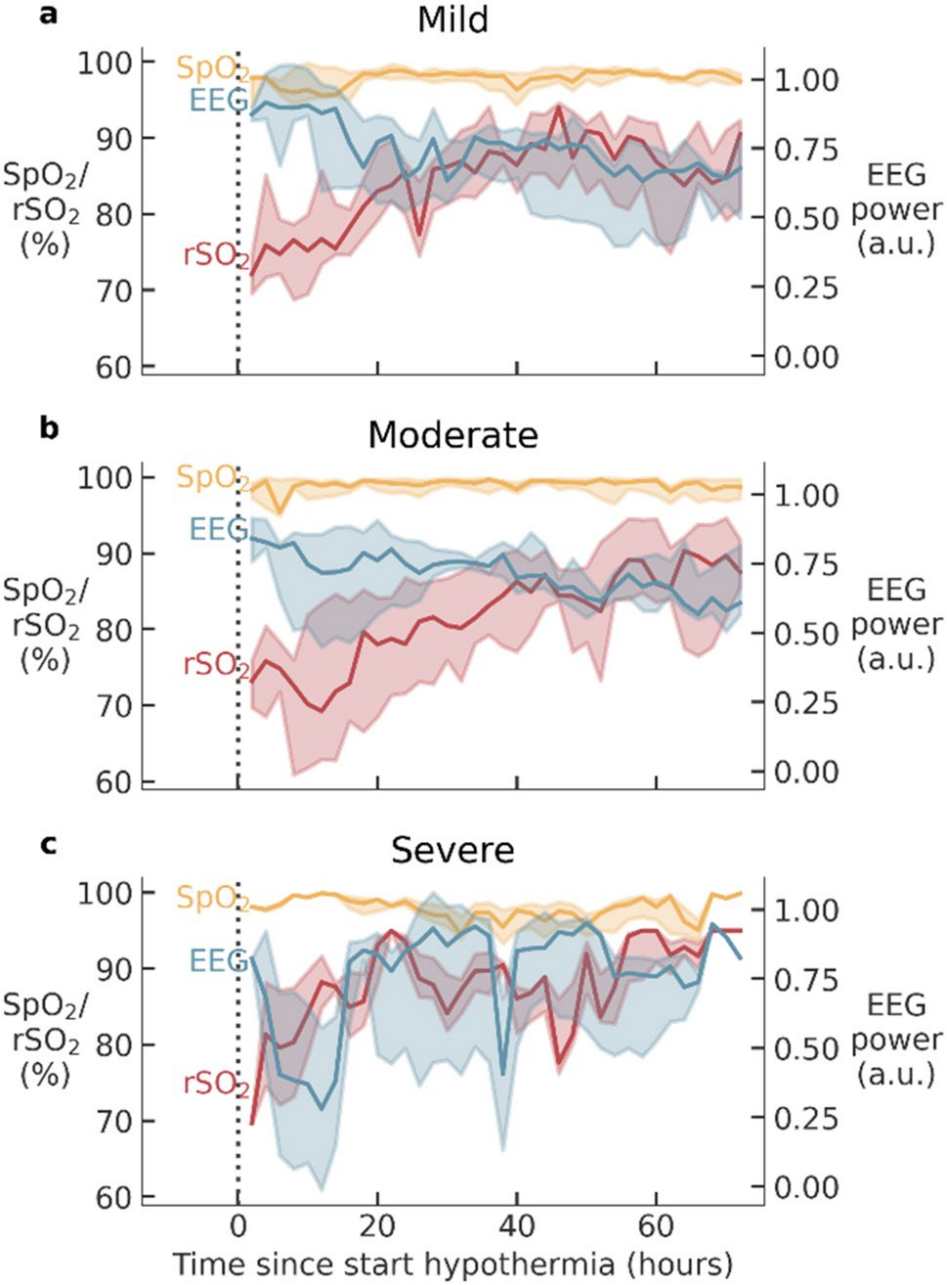
Hourly average values for EEG log bandpower, rSO2 and SpO2 per outcome group. (**a**) Mild outcome, (**b**) moderate outcome, (**c**) severe outcome. Solid lines represent the median and the shaded areas the inter-quartile range. The vertical dashed line represents the start of hypothermia.

EEG, rSO2 and SpO2 signals recorded during 72 h of hypothermia were extracted, although data was not always available for the full period. The median duration of the extracted recordings and their inter-quartile range (IQR) was 68.8 (64.1–71.8) h. The signals were synchronized on onset time of hypothermia and are visualized per outcome group in Fig. 1, where time 0 refers to the start of hypothermia. A clear increasing trend is visible in rSO2 values, starting at approximately 70% and gradually increasing up to 90%. A similar evolution is observed for the mild and moderate groups, while the increase in rSO2 seems to be faster in the severe outcome group, reaching rSO2 values > 90% in the first day of cooling. A weak decreasing trend is visible in EEG power during the cooling period in the mild and moderate group. In the first day of cooling (0–24 h), the EEG power of the severe group is lower compared to EEG power in the mild and moderate groups. SpO2 remained close to 100% in all groups.

### Partial wavelet coherence

**Figure 2:**
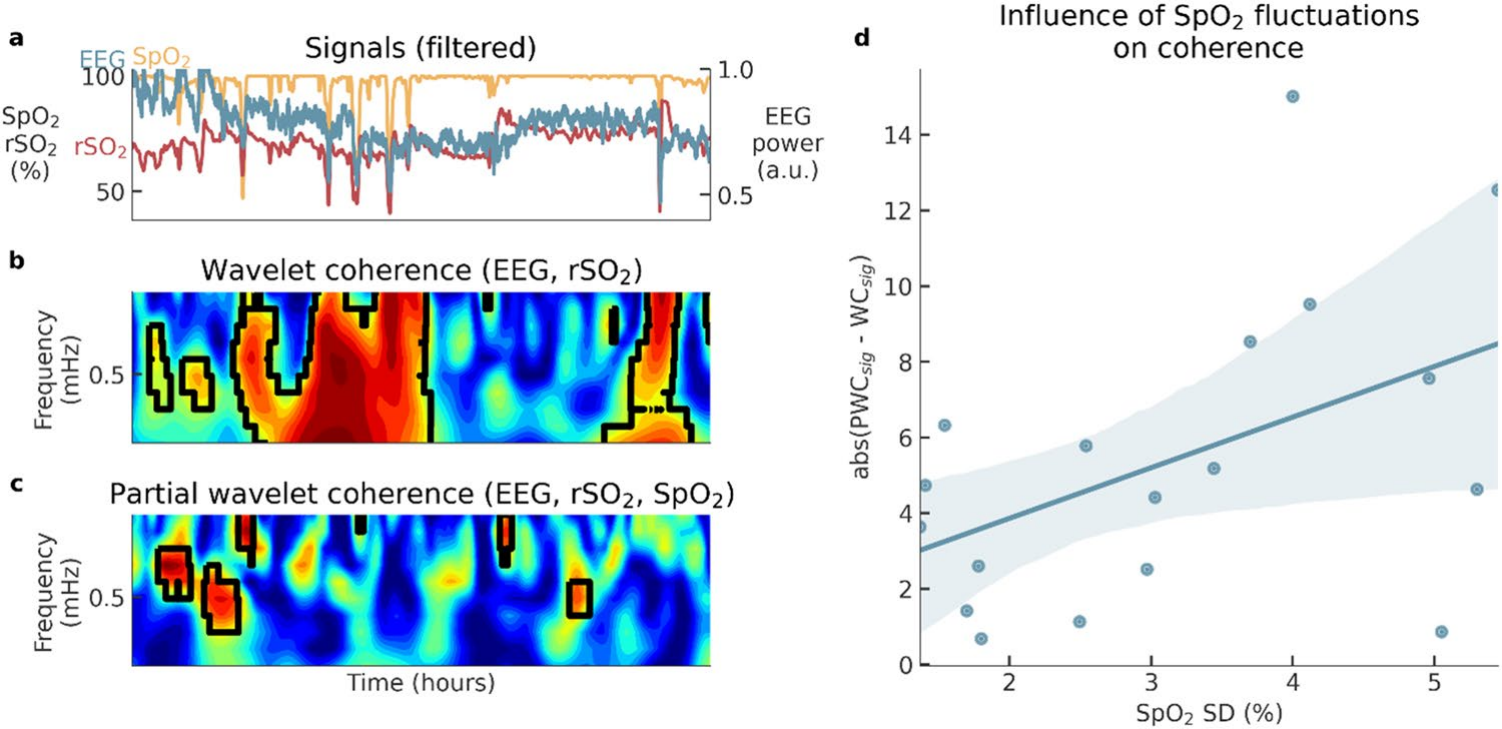
Comparison of regular wavelet coherence and partial wavelet coherence. (**a**) Input signals are smoothed with a 5-min moving average to correspond with the frequency range illustrated in the wavelet coherence. (**b**) Regular wavelet coherence between EEG and rSO2. (**c**) Partial wavelet coherence removes the influence of the SpO2 signal. Regions with significant coherence are encapsulated by black contours. (**d**) The relationship between variability (standard deviation) of SpO2 and the difference between regular wavelet coherence and partial wavelet coherence (percentage of significant coherence).

Figure 2 illustrates the effect of applying partial wavelet coherence compared to regular wavelet coherence. In the top left figure, it is clear that the EEG, rSO2 and SpO2 signals are all strongly correlated. In the middle left plot, regular wavelet coherence identifies large ‘blobs’ of significant coherence between EEG and rSO2 as indicated by the dark red areas. In contrast, much less significant coherence is detected by PWC (bottom left), due to the correction for confounding changes in SpO2. Moreover, by removing the influence of SpO2, new fluctuations are discovered with PWC. The plot on the right shows the relation between the standard deviation of SpO2 and the difference between regular and partial wavelet coherence in terms of total percentage of significant coherence. As expected, the difference between the coherence methods increases as the variability of SpO2 increases.

**Figure 3:**
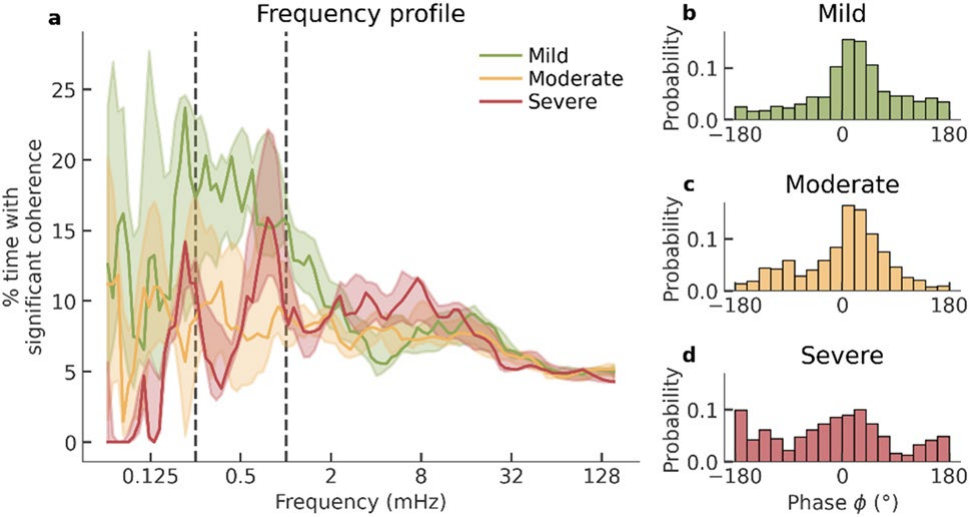
Frequency and phase of partial wavelet coherence. (**a**) Frequency profiles for each outcome group computed as time average coherence during 72 h of hypothermia. The solid line represents median per group and the shaded areas represent the inter-quartile ranges. The vertical dashed lines indicate the frequency band of interest (0.25–1 mHz). (**b**–**d**) distribution of the phase of the significant coherence in the 0.25–1 mHz band per outcome group. $\phi \approx 0^\circ$ indicates in-phase coherence.

From the PWC time–frequency maps represented in Fig. 2, frequency profiles are computed by determining the percentage of time with significant coherence per frequency (including all phases). The left graph in Fig. 3 shows these frequency profiles per outcome group for the entire cooling period (0–72 h). Frequency profiles for other time periods were similar and can be found in section S2 of the supplementary material. Most coherence was found at very low frequencies (0.25–1 mHz) and was higher in the mild group compared to the moderate and severe groups. The three plots on the right in Fig. 3 show the distributions of the phase angle of all significant PWC values. In the mild and moderate group, most coherence is in-phase ($\phi \approx 0^\circ$), whereas both in-phase and anti-phase coherence were dominant in the severe group.

### Comparison with traditional biomarkers

**Figure 4:**
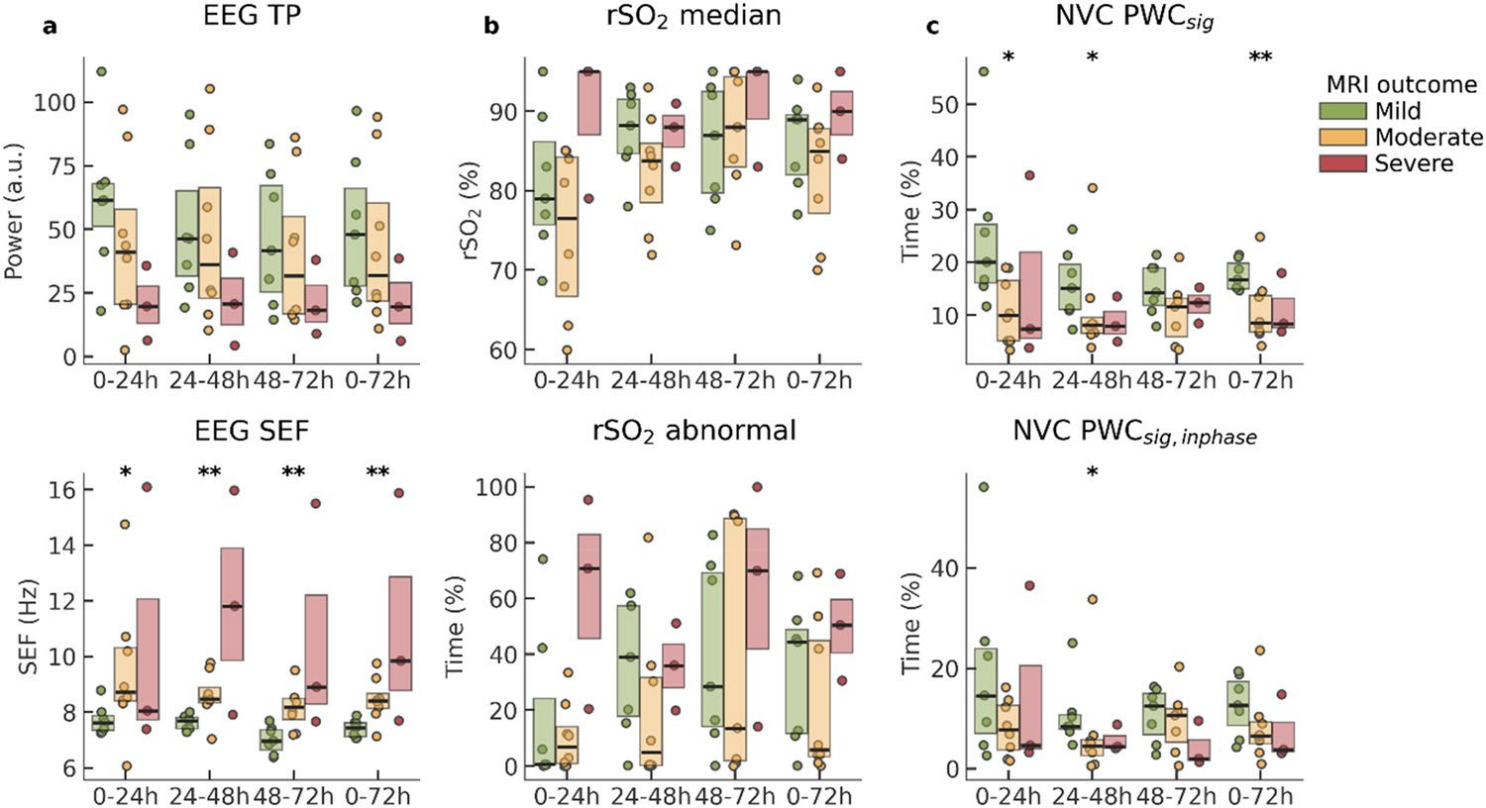
Biomarkers per outcome group per analysing window. (**a**) EEG biomarkers, (**b**) NIRS biomarkers, (**c**) NVC biomarkers. Hours are relative to the start of hypothermia. Asterisks indicate a significant difference (* p < 0.05, ** p < 0.01) between the mild vs the pooled moderate and severe group, using a Mann–Whitney U test. EEG TP: EEG total power (0.5–19.5 Hz), EEG SEF: EEG spectral edge frequency, rSO2 median: median rSO2 value, rSO2 abnormal: percentage of time where rSO2 is outside the normal range (55–90%), NVC PWC_sig_: percentage of time with significant coherence (all phases) in 0.25–1 mHz, NVC PWC_sig,inphase_: percentage of time with significant in-phase ($\left|\phi \right|<{90}^{o}$) coherence in 0.25–1 mHz.

Figure 4 shows how EEG, rSO2 and NVC biomarkers differ between outcome groups. EEG spectral edge frequency (EEG SEF) and NVC features show significant differences when comparing mild to moderate and severe outcome groups. These differences are visible in all 3 days of cooling, though not always significant for the NVC features. Total EEG power (TP) does not show significant differences, but tends to be highest in the mild group and lowest in the severe group, indicating suppressed EEG activity in neonates with adverse outcome. Furthermore, rSO2 tends to have higher and more abnormal values in the severe group, especially in the first 24 h of TH. We refer to section S1 in the supplementary material for a similar plot with results obtained with regular wavelet coherence. Those results have similar trends as partial wavelet coherence, but the differences between the outcome groups are smaller and not significant.

**Figure 5:**
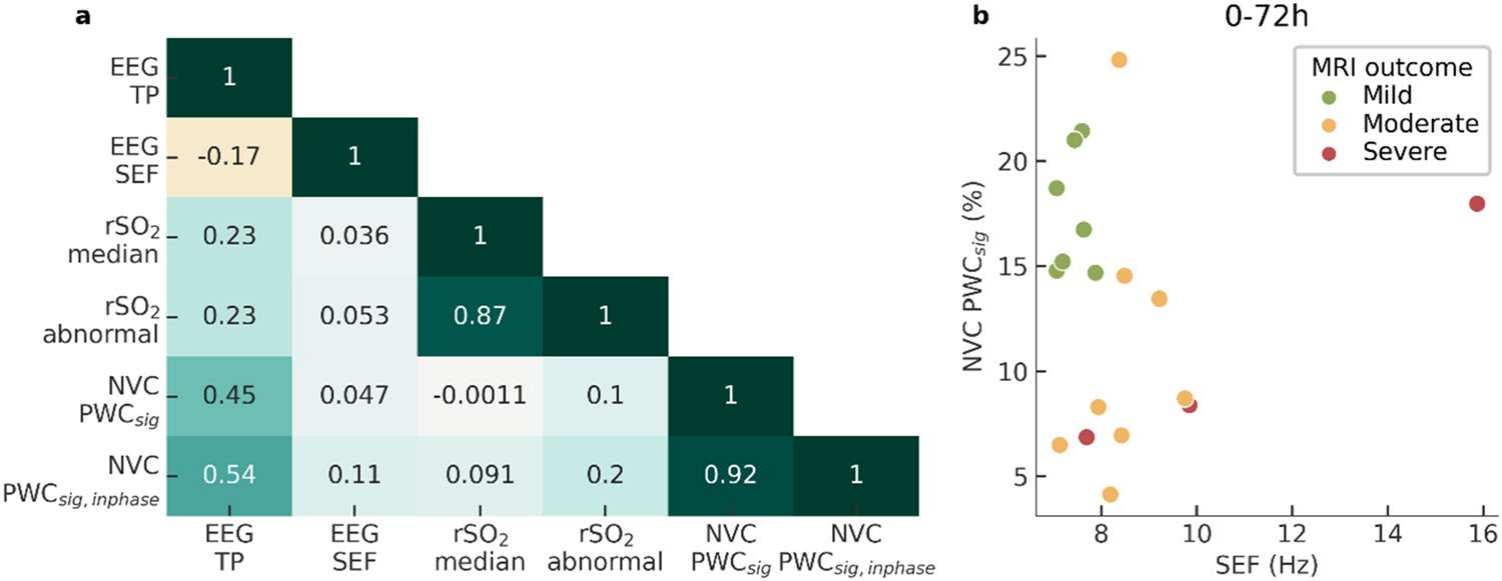
Relation of NVC biomarkers to EEG and NIRS biomarkers. (**a**) Correlation heat map between the features computed in the total time window (0–72 h). (**b**) EEG SEF and NVC PWC_sig_ provide complementary information to identify neonates with mild MRI outcome.

The left panel of Fig. 5 shows a correlation heat map between the biomarkers from different modalities, computed in the total cooling period (0–72 h). The low correlation coefficients between NVC features and the EEG and rSO2 features indicate that the NVC features provide complementary information. Since Fig. 4 suggest that EEG SEF and PWC_sig_ are the most promising features, we illustrate their combined use in the right scatterplot of Fig. 5. This scatterplot visually enhances the representation of the combined two biomarkers. A low PWCsig or low SEF characterizes neonates with a moderate or severe MRI outcome.

## Discussion

From this study a novel method for quantification of NVC using continuous EEG, rSO2 and SpO2 recordings was proposed correcting for confounding changes in SpO2. This use of partial wavelet coherence (PWC) method builds further on the current state-of-the art wavelet method and eliminate the confounding effect of SpO2. Furthermore, the study compared the NVC biomarkers obtained with PWC to traditional quantitative EEG and NIRS features. To our knowledge, this is the first study to remove confounding effects of SpO2 in NVC using wavelet coherence methods, as well as the first study to compare the predictive value of NVC biomarkers with traditional EEG and NIRS biomarkers.

PWC is the wavelet equivalent of partial correlation and effectively removes coherence caused by confounding changes in SpO2 as illustrated in Fig. 2. Removal of the effect of SpO2 is important for interpreting the PWC as a measure for NVC for two main reasons. Firstly, NVC describes regulation of CBF in response to cerebral metabolic activity and due to the strong dependence of rSO2 on SpO2, rSO2 is only a valid surrogate for CBF under the assumption of stable SpO2^33^. Secondly, besides affecting rSO2 levels, decreased levels of SpO2 can also drive changes in EEG as a result of decreased energy supply, thus potentially introducing spurious coupling between rSO2 and EEG not driven by NVC, but by SpO2. For these two reasons, coupling between EEG and rSO2 should only be considered as a measure for NVC while SpO2 is stable or while controlling for changes in SpO2. Although SpO2 is typically constant for the majority of time, arterial desaturations are inevitable in long recordings, especially in sick neonates. We therefore suggest using PWC to eliminate the effect of SpO2. If SpO2 is relatively stable, PWC will yields similar results as regular wavelet coherence, while it corrects the coherence estimates if SpO2 is less stable (see Fig. 2).

With PWC, the correction for SpO2 is incorporated in the computation of the coupling, as local linear relationships between SpO2 and EEG and rSO2 are removed in time–frequency space^36^. This is different from methods that remove the influence of SpO2 on rSO2 as a pre-processing routine, such as the one proposed by Caicedo et al.^34^. Even though those methods that correct for SpO2 as a pre-processing routine are more general and can be combined with any mathematical method for detecting coupling, their downside is that they assume or estimate a model between SpO2 and the other variables to compute the correction. In contrast, by incorporating the elimination of SpO2 influences in the computation of the coupling using PWC, no additional model needs to be considered for this correction. PWC can also be extended to correct for more than one confounding variable, as described by Meng^38^. In the context of NVC, this extension of PWC can for example be used to simultaneously correct for changes SpO2 and mean arterial blood pressure (MABP). Discussing the effect of variations in MABP is out of the scope of this study, but preliminary results showed that adding MABP to the confounding variables in PWC did not change the results presented in this paper. We included these preliminary results in section S3 of the supplementary material.

Most coherence was found in the very low frequencies (< 2 mHz, see Fig. 3). The observed coherence in 0.25–1 mHz means that there are common fluctuations in EEG power and rSO2 with cycle times ranging from 17 to 67 min. We found that the amount of significant (in-phase) coherence between 0.25 and 1 mHz differed per outcome group (Fig. 4). This is in keeping with findings by Chalak et al.^25^ who looked at wavelet coherence between amplitude-integrated EEG (aEEG) and rSO2 and found that the amount of in-phase coherence between 0.25 and 1 mHz was related to long-term developmental outcome. Similarly, Das et al.^26^ found that coherence between aEEG and rSO2 in very low frequencies is predictive for short-term brain injury as visible on MRI. Our outcomes were similar however with two important differences. Firstly, Chalak and Das et al., used regular wavelet coherence and not partial wavelet coherence used in this study. The effect of using regular wavelet coherence versus partial wavelet coherence is related to the instabilities in arterial oxygen saturation. The more stable the SpO2 is, the more the regular and partial coherence will agree, which explains the correlation between the results of those methods. Nevertheless, as argued before, we suggest that PWC provides a more robust method and analysis outcome. Moreover, we noticed that the outcome groups were clearly more heterogeneous when using partial coherence compared to regular wavelet coherence, as illustrated in section S1 of the supplementary material. Secondly, whereas Chalak and Das et al., used aEEG bandwidth, we chose to compute EEG power, which is a simpler and more intuitive measure of brain activity and has been linked to cerebral metabolism in various studies of neonatal HIE^8,17,49,50^. Moreover, preliminary results showed that more coherence was detected using EEG power, indicating a higher sensitivity.

NVC as characterized by PWC_sig_ and PWC_sig,inphase_ was higher in the mild outcome group compared to moderate and severe groups (Fig. 4). This difference was significant during the periods 0–24 h, 24–48 h and 0–72 h for PWC_sig_ and for 24–48 h for PWC_sig,inphase,_ while the trend was consistent among all time windows. These results are in agreement with Das et al.^26^ who reported a decreased amount of wavelet-based NVC in the 24 h of life in neonates with brain injury. The NIRS features were not significantly predictive of MRI outcome, although high rSO2 values during day 1 of cooling seem to be indicative of severe MRI outcome. This is in line with previous studies that report a relationship between severe outcome and elevated rSO2 values in the first day of life, which might be explained by reduced cerebral oxygen consumption by the injured brain^12,17,20,51^. EEG features showed more pronounced differences between outcome groups than NIRS, as reported in previous research^14,17–19^. EEG SEF was especially significantly lower for neonates with mild MRI outcome compared to moderate and severe outcome. This is in line with the results of Lacan et al.^6^ who reported a correlation between increased SEF and worse visual EEG grades, as well as with Thordstein et al.^52^, who reported increased levels of SEF in asphyxiated infants compared to healthy controls. On the other hand, our results conflict with the work of Garvey et al.^53^ who reported lower SEF in neonates with mild HIE (non-cooled) compared to healthy controls. A possible explanation for the discrepancies between these studies is that their study design and methods may differ, including their selection criteria of patients and outcome measures and criteria. Furthermore, specific pre-processing and algorithmic choices, such as filter specification and segment lengths, differ among these studies which is known to influence the computed features^54^. Direct comparisons between studies is therefore difficult. Further investigation of our results confirmed that EEG SEF negatively correlated with relative EEG delta power, i.e., the lower SEF values found in the mild group can be explained by increased relative delta power in this group. In turn, increased delta power has been associated with favourable outcome in neonates with HIE in various other studies, supporting our findings for EEG SEF^8,49,52,55^.

To investigate the added value of the proposed NVC biomarkers to existing EEG and NIRS biomarkers, correlations were computed between all biomarkers, as visualized in Fig. 5. The correlations were generally low, suggesting that NVC provides novel information, complementary to existing EEG and NIRS features. This notion is further strengthened by the graph on the right in Fig. 5, which shows that the combined use of SEF EEG and NVC PWC_sig_ can isolate the mild group from the moderate and severe groups. Nevertheless, we observed a moderate correlation of 0.5 between NVC features and total EEG power (EEG TP). This is not surprising as less variation and hence coherence with rSO2 can be expected in suppressed EEG. Despite their moderate correlation, the predictive power of NVC features seems to be larger than for EEG TP, as evident from Fig. 4, indicating that NVC could be a more useful feature than EEG TP.

Although the total significant coherence computed for all phases (PWC_sig_) seems to separate the outcome groups better than the significant in-phase coherence (PWC_sig,inphase_), the latter is driving the observed values for PWC_sig_. This is evident from Figs. 3 and 5 that show that significant PWC was predominantly in-phase ($\phi \approx 0$) and that there is a high correlation between PWC_sig_ and PWC_sig,inphase_. We hypothesize that in-phase PWC especially can be interpreted as a measure of NVC, since a simultaneous increase of EEG power and rSO2 can best be explained by an increase in CBF^24^. For illustration, consider the following situation. If cerebral activity increases, the brain utilizes more oxygen and hence the venous oxygen saturation decreases. Without any changes in CBF, this decrease in venous oxygen saturation would lead to a decreased rSO2. This situation would manifest as anti-phase coherence, since a decrease rSO2 is occurring simultaneously with an increase in EEG power. However, in our results we notice primarily in-phase coherence, which indicates a simultaneous increase in both rSO2 and EEG power. We hypothesize that such a simultaneous increase in rSO2 and EEG power can be explained by an increase in CBF, overcompensating the initial decrease in rSO2 due to increased oxygen consumption. An increase in CBF in relation to increased cerebral activity suggests active regulation of CBF in response to cerebral activity, which indicates intact NVC. Therefore, we hypothesize that high in-phase coherence can be interpreted as a marker for NVC. On the contrary, we hypothesize that reduced in-phase coherence can be explained either by impaired NVC, or a lack of cerebral activity which is common in neonates with extreme hypoxia. In the latter case, the lack of cerebral activity might reduce spontaneous changes in EEG activity, thereby not triggering NVC.

Despite this motivation for focusing on in-phase coherence, we observed that the total significant coherence across all phases (PWC_sig_) seems to be more sensitive than PWC_sig,inphase_. Our hypothesis is that coherence in general (without looking at the phase) can be expected to be lower in neonates with less EEG or rSO2 variability. Furthermore, the INVOS NIRS neonatal sensor has limitations, with rSO2 values clipped at 95%. No variation therefore exists in the recorded rSO2 signal if rSO2 > 95%, which occurs more often in neonates with adverse outcome, supposedly as result of decreased cerebral oxygen consumption. Taking this into account, since suppressed EEGs and elevated (> 95%) rSO2 are related to adverse outcome, their decreased variability might reduce PWC_sig_, making PWC_sig_ even more sensitive to MRI outcome than PWC_sig,inphase_.

Besides (partial) wavelet coherence, various other methods for quantification of EEG-NIRS coupling have been used outside of the context of HIE, such as the radial basis function kernel and (nonlinear) transfer entropy^27,29,30,32^. Each of these methods make different assumptions about the timescale and complexity of the coupling and may therefore capture a different component of NVC. Apart from the high interpretability, we consider two more general advantages of wavelet coherence. Firstly, the intrinsic time–frequency decomposition of wavelet coherence provides an elegant framework for analysing non-stationary interactions between EEG and NIRS at multiple frequencies, allowing one to disentangle and study the coupling at multiple timescales simultaneously. Secondly, the phase angle obtained by wavelet coherence provides an estimate of the nature of the coupling (positive vs negative correlation) which we hypothesize to be important information for the interpretation of EEG-rSO2 coupling, as discussed above. Due to the multiscale nature of PWC, we were able to conclude that there is significant coupling in the very low frequency range, i.e., at very large timescales, whereas there seems to be no evidence for coupling at shorter time scales. Hendrikx et al.^30^ however, showed that nonlinear transfer entropy is able to detect interaction between EEG and NIRS at shorter time scales (max 15 min). Therefore, we reason that quantification of NVC should not be limited to one type of method as several different methods might provide additional pieces of information.

The type of NVC described in this paper is different from the classical definition of NVC. Whereas the latter refers to a local link between neural activity and cerebral blood flow, in this study we reported a more global coupling that acts at much larger time scales. The set-up that was used in this study is not well suited for studying local NVC, as the EEG and NIRS systems do not cover the same brain areas. Nevertheless, we assume that the obtained EEG and NIRS signals in our study adequately reflect global changes in cerebral activity and oxygenation, thereby making them suited for studying global NVC. For studying NVC at a more local level and describing the regional differences, a multimodal cap with EEG and NIRS electrodes, such as the one recently described by Frijia et al.^56^ could be used. However, such set-ups are not easy to be used in a daily clinical setting. Therefore, we opted for the more feasible method using C3–C4 EEG and NIRS placed on the forehead. Additionally, an advantage of using C3–C4 is that this channel is also available when only a cerebral function monitor (CFM) is used (instead of full EEG), which is often the case in neonatal units.

The observed coherence in 0.25–1 mHz relates to simultaneous oscillations of EEG power and rSO2 with time periods of 17–67 min. When trying to establish a physiological origin of these coupled dynamics, one could argue that this might be related to sleep cycling, since the time periods of those dynamics are similar to the duration of sleep cycles in neonates at term age. Moreover, cyclic patterns in EEG power and rSO2 are known to be related to sleep cycling^55,57^. We did not however study whether the coherence was related to sleep cycling, as all neonates were sedated and typically did not show normal sleep cycling during cooling. Additionally, Chalak et al., did not observe any relation with sleep staging in their patient cohort. Nevertheless, despite the absence of normal, typical sleep cycling, there might be underlying cyclic patterns in brain activity, that could potentially be related to sleep staging. More research needs to be done to explore the exact biological understanding of the observed EEG-rSO2 coupling, which can further increase the interpretation and validity of the proposed biomarkers.

Limitations of this study include a small patient group and unknown effects of sedative, inotropic and anti-epileptic drugs. Administered drugs differed for each neonate according to the clinical need. Since the typically administered drugs are known to affect brain activity and/or (cerebral) blood flow, drugs might affect the (estimation of) NVC. Future research should therefore focus on validation of wavelet coherence methods in larger patient cohorts as well as on studying the effects of medication on wavelet-based NVC quantification.

## Conclusion

We proposed the use of partial wavelet coherence to eliminate the effect of confounding changes of SpO2 on coupling between EEG and rSO2 for the assessment of NVC in neonates with HIE undergoing TH. This novel correction for changes in SpO2 increases the robustness and interpretability of automated NVC analysis in long-term recordings. We further validated the clinical value of an automated wavelet-based biomarker for NVC in neonates with HIE and showed that the NVC biomarkers obtained with partial wavelet coherence provide novel prognostic information, additional to traditional individual EEG and NIRS features, where decreased levels of NVC were related to worse MRI outcome.

## Supplementary Information

Supplementary Information.

## Data Availability

All computations and analyses were done using custom Python code which is publicly available at https://gitlab.com/timhermans/pwc.

## Data availability

The datasets analysed during the current study are not publicly available due to their sensitive nature but are available from the corresponding author on reasonable request.
