# Supplementary material for: Partial wavelet coherence as a robust method for assessment of neurovascular coupling in neonates with hypoxic ischemic encephalopathy
Source: Sci Rep. 2023 Jan 10;13:457. doi: 10.1038/s41598-022-27275-8 (PMC9832127; doi:10.1038/s41598-022-27275-8)
Supplement: Supplementary file 1 — Supplementary Information. [file 41598_2022_27275_MOESM1_ESM.pdf]

Partial wavelet coherence as a robust method for assessment of neurovascular coupling in neonates with hypoxic ischemic encephalopathy

Tim Hermans*[a], Katherine Carkeek[b,c,d], Anneleen Dereymaeker[b,c], Katrien Jansen[b,e], Gunnar Naulaers[b,c], Sabine Van Huffel[a] and Maarten De Vos[a,b]

[a] Department of Electrical Engineering (ESAT), STADIUS, KU Leuven, Belgium
[b] Department of Development and Regeneration, KU Leuven, Belgium
[c] Neonatal Intensive Care Unit, UZ Leuven, Belgium
[e] Child Neurology, UZ Leuven, Belgium
[d] Neonatal Intensive Care Unit, Cliniques Universitaires Saint Luc, Belgium
*Corresponding author.
Email: tim.hermans@esat.kuleuven.be

Supplementary material

S1 Wavelet coherence vs partial wavelet coherence

Figure S1 compares the NVC biomarkers computed with regular wavelet coherence and partial wavelet coherence. The partial wavelet coherence removes the confounding influence that arterial oxygen saturation has on EEG power and rSO2 values. By removing this influence the NVC features better separate the MRI outcome groups, suggesting that instabilities of SpO2 in some patients can negatively affect the estimation of EEG-rSO2 coupling.

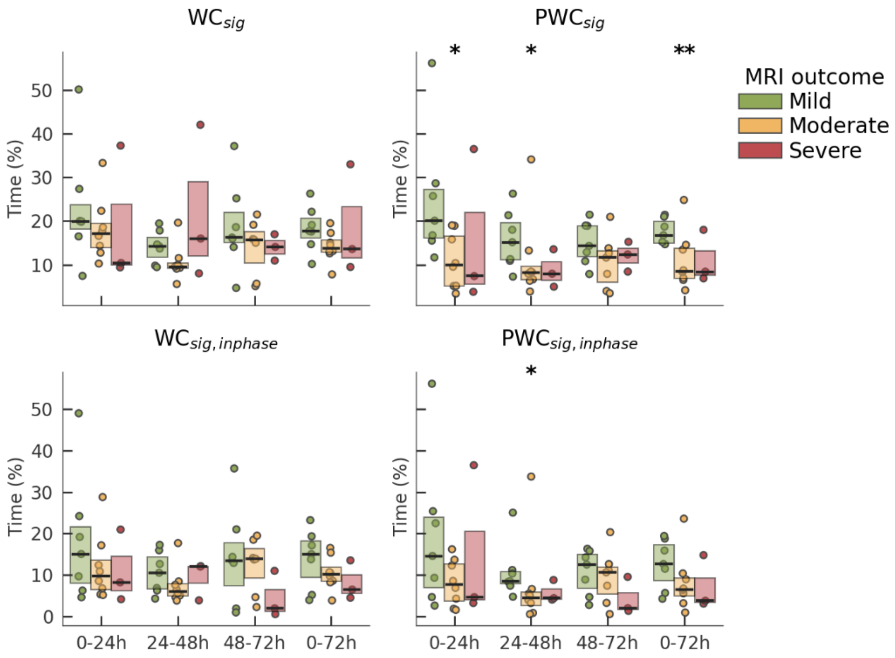

***Figure S1** Comparison of NVC biomarkers computed with regular wavelet transform (left) and partial wavelet transform correcting for confounding SpO2 variations (right).*

S2 Partial wavelet coherence in different time windows

Figure S2 shows percentage of time with significant PWC (PWC_{sig}), as well as the percentage of time with significant inphase ($|\phi| < 90°$) PWC computed ($PWC_{sig,inphase}$) per frequency computed in different periods. Figure S3 shows the frequency profiles and relation between NVC biomarkers, EEG SEF and MRI outcome for different periods during cooling. Despite EEG and rSO2 signals are subject to change/development during cooling, these figures show that the NVC results are relatively stable across the different time periods (days of cooling).

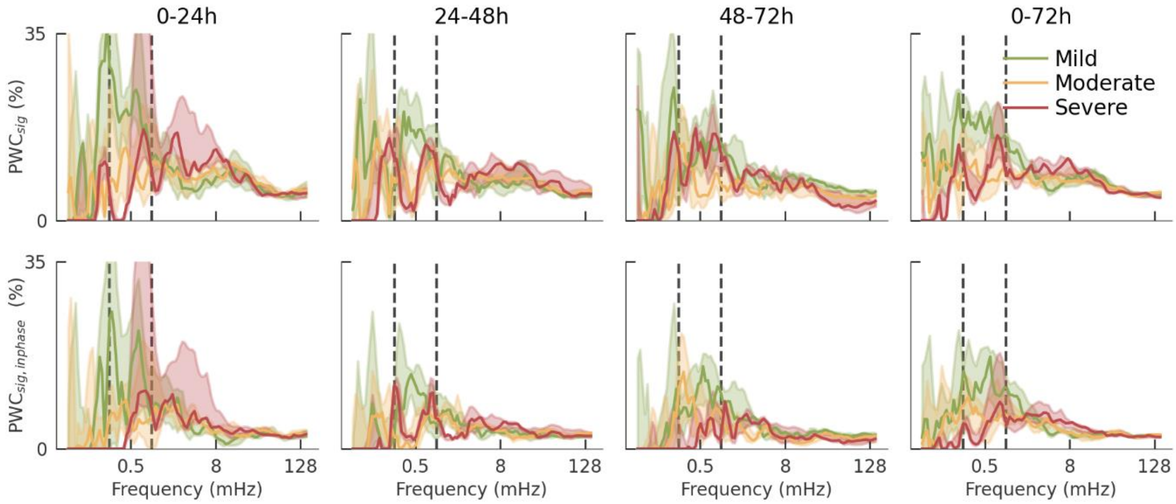

Figure S2 *Frequency profiles showing the percentage of significant partial wavelet coherence per day. Top: total significant coherence (all phases), bottom: inphase coherence ($|\phi| < 90°$). The dashed vertical lines indicate the 0.25-1 mHz frequency band.*

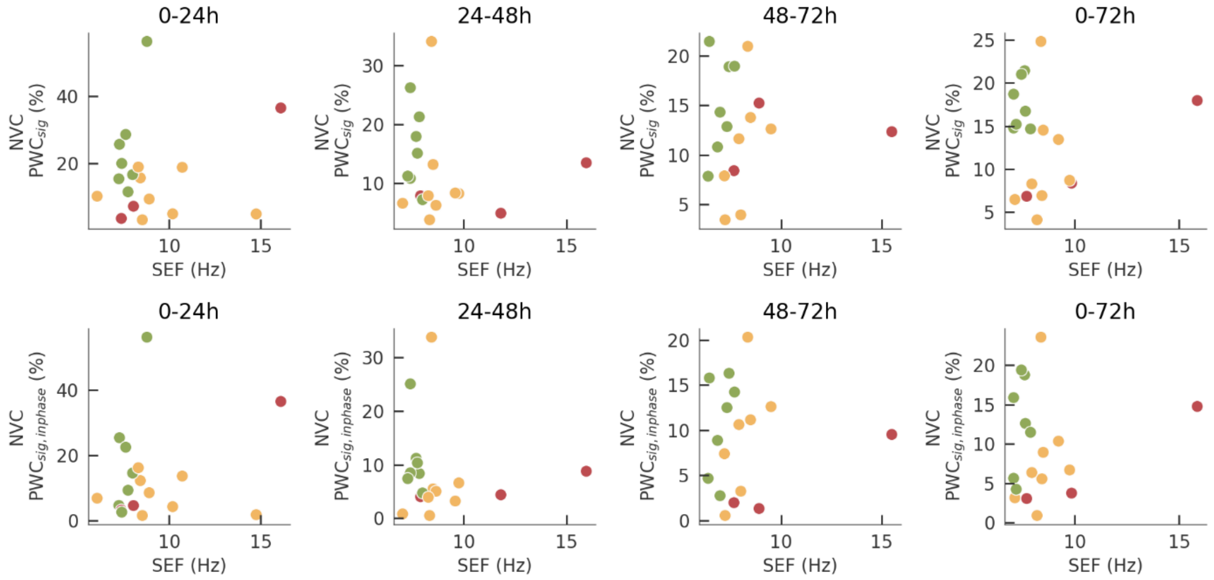

Figure S3 *Scatterplot of EEG spectral edge frequency (SEF) and percentage of time with significant coherence between 0.25-1 mHz per time window, color-coded by outcome group. Top: total significant coherence (all phases), bottom: inphase coherence ($|\phi| < 90°$).*

S3 Mean arterial blood pressure as a second confounding variable

Mean arterial blood pressure (MABP) could be considered as another confounding factor. To investigate the importance of MABP for assessment of NVC, we computed MABP-EEG and MABP-rSO2 wavelet coherence and observed that MABP is coupled with both EEG and rSO2 (see Figs. S4 and S5).

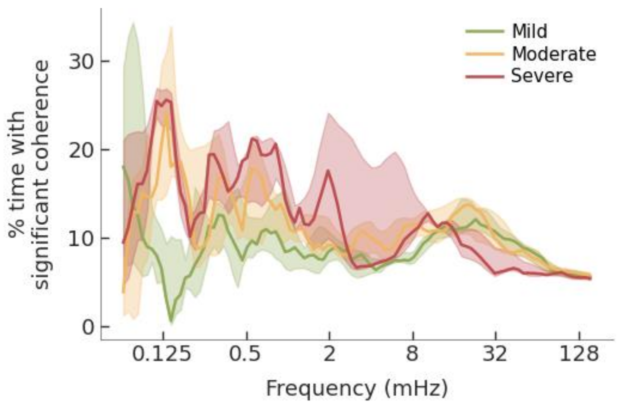

Figure S4 *Frequency profiles of the wavelet coherence between MABP and EEG.*

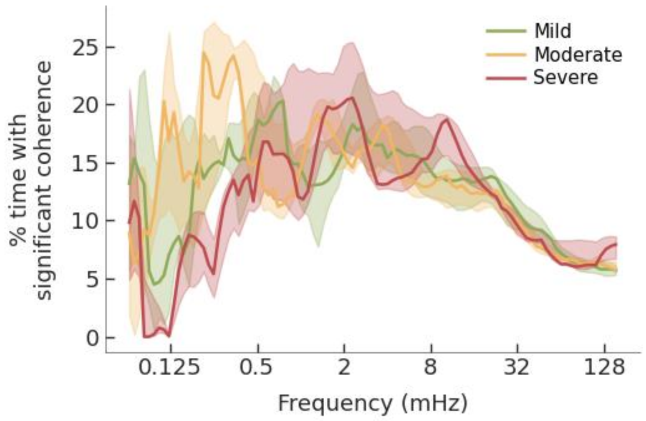

Figure S5 *Frequency profiles of the wavelet coherence between MABP and rSO2.*

To remove the potential confounding influence of MABP on the coupling between EEG-rSO2, we used the extension of partial wavelet coherence that allows to correct for multiple confounding variables as described by Meng [1]. With this method we can compute the coupling between EEG and rSO2, while correcting for confounding effects of both SpO2 and MABP (see Figure S6). The obtained results with the added correction for MABP are very similar to the original results presented in the paper (see Figure 3 in the paper). Additionally, the effect on the NVC features as presented in Figure S7 is small compared to the ones presented in the paper. One potential explanation for the absence of a confounding effect of MABP on EEG-rSO2 coupling could be that MABP seems to be coupled with EEG and rSO2 at different frequencies, as can be seen in the frequency profiles of the MABP-EEG and MABP-rSO2 coherence (Figs 1 and 2 below).

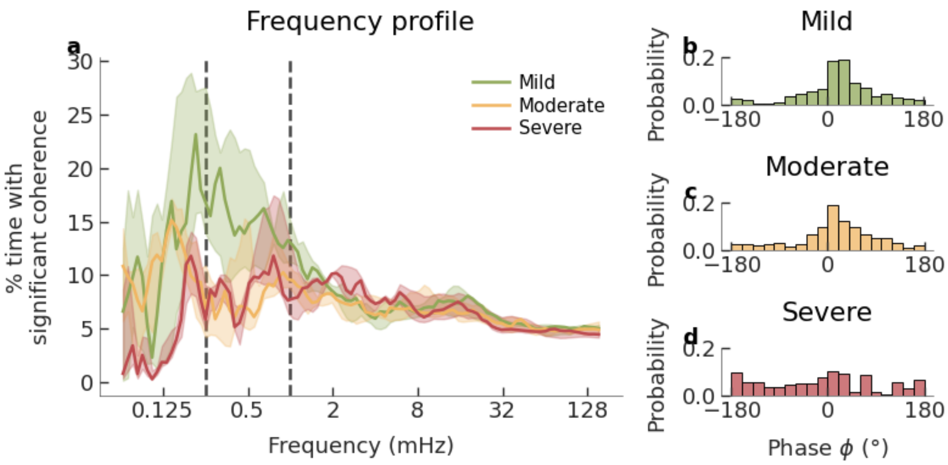

Figure S6 *Frequency profiles and the distribution of the phase angles in the 0.25-1 mHz range of partial wavelet coherence between EEG and rSO2, correcting for confounding changes in SpO2 and MABP.*

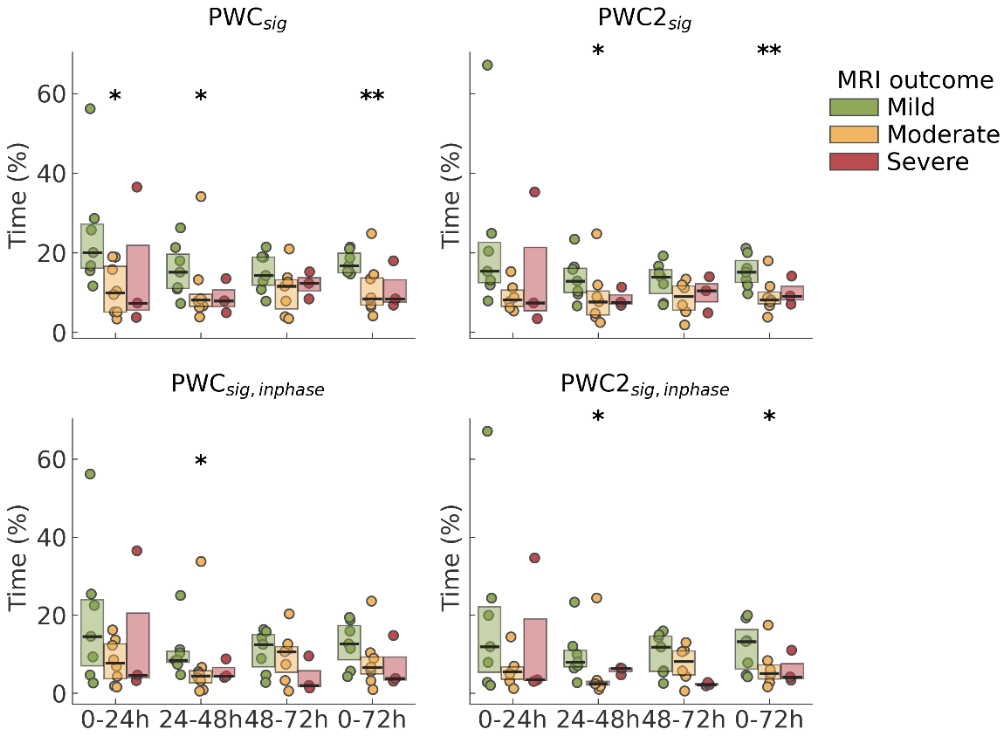

Figure S7 *Comparison of the partial wavelet coherence features computed with SpO2 as the only confounding variable (PWC, left, same as in the paper) vs SpO2 and MABP as two confounding variables (PWC2, right).*

References

1. Meng, X. The time-frequency dependence of unemployment on real input prices: a wavelet coherency and partial coherency approach. *Applied Economics* **52**, 1124–1140 (2020).
